# The association between platelet indices and presence and severity of psoriasis: a systematic review and meta-analysis

**DOI:** 10.1007/s10238-022-00820-5

**Published:** 2022-04-04

**Authors:** Z. Liu, L. A. Perry, V. Morgan

**Affiliations:** 1 Department of Dermatology, The Royal Melbourne Hospital, Parkville, Australia; 2 Department of Medicine, Melbourne Medical School, Faculty of Medicine, Dentistry and Health Sciences, The University of Melbourne, Parkville, Australia; 3 Department of Anaesthesia, The Royal Melbourne Hospital, Parkville, Australia; 4 Department of Critical Care, Melbourne Medical School, Faculty of Medicine, Dentistry and Health Sciences, The University of Melbourne, Parkville, Australia

**Keywords:** Psoriasis, Platelets, Systematic review, Meta-analysis

## Abstract

The role of platelet function indices—platelet count (PLT), mean platelet volume (MPV), platelet distribution width (PDW), plateletcrit (PCT), immature platelet fraction (IPF), and platelet mass index (PMI)—in psoriasis is uncertain. This systematic review and meta-analysis aimed to evaluate the association of these platelet biomarkers with both presence and severity of psoriasis. We searched MEDLINE (Ovid), Embase (Ovid), and the Cochrane Library from inception to November 2021. To evaluate the association of platelet function indices and psoriasis, we recorded mean differences (MD) and 95% confidence intervals (CI) as well as correlation coefficients (r) for each included study, and generated summary estimates using random-effects inverse-variance modelling. We screened 1,079 unique studies, and included 33 studies with 6724 patients in the quantitative analyses. Compared with controls, patients with psoriasis had higher PLT (MD 12.86 × 10^9^/L, 95% CI 6.34–19.39, *p* < 0.001), MPV (MD 0.61fL, 95% CI 0.31–0.92, *p* < 0.001), and PCT (MD 0.05%, 95% CI 0.01–0.09, *p* = 0.010), but similar PDW (MD 0.16%, 95% CI -0.46–0.79, *p* = 0.610). Psoriasis Area and Severity Index (PASI) was weakly correlated with PLT (r 0.17, 95% CI 0.06–0.28, *p* = 0.003), MPV (*r* 0.36, 95% CI 0.22–0.49, *p* < 0.001), and PDW (*r* 0.17, 95% CI 0.08–0.26, *p* < 0.001). Study numbers were insufficient to judge the relationship of IPF and PMI with psoriasis presence, or PCT, IPF, and PMI with psoriasis severity. In summary, PLT, MPV, and PCT are significantly elevated in patients with psoriasis, and PLT, MPV, and PDW are weakly correlated with PASI. Future studies are needed to evaluate the independent diagnostic and prognostic potentials of these biomarkers in patients with psoriasis.

**Supplementary Information:**

The online version contains supplementary material available at 10.1007/s10238-022-00820-5.

## Introduction

Psoriasis is a common chronic inflammatory skin disorder with a variable worldwide prevalence of up to 11.4% in adults and 1.4% in children [1]. Although classically associated with the development of multiple inflammatory skin plaques in an extensor distribution, a growing body of evidence now substantiates an appreciation of psoriasis as a systemic disease with associated extracutaneous comorbidities [2]. In particular, patients with psoriasis are more likely to exhibit comorbid atherosclerotic vascular disease—cardiovascular, cerebrovascular, and peripheral vascular—and its risk factors such as obesity, metabolic syndrome, hypertension, diabetes, and smoking [3, 4].

The underlying pathogenesis of psoriasis and its relationship to cardiovascular disease remains incompletely understood. The association between platelet activation and cardiovascular disease is known [5–7]. More recently, studies have investigated the association between platelet activation and psoriasis [8, 9], reflecting an emerging sphere of research which aims to investigate the diagnostic and prognostic potentials of simple blood parameters in the context of increasing access to, cost-effectiveness, and routinisation of haematological assays [10–15]. Specifically, platelet count (PLT), mean platelet volume (MPV), platelet distribution width (PDW), plateletcrit (PCT), immature platelet fraction (IPF), and platelet mass index (PMI) are haematological markers of platelet function which have demonstrated associations with both cardiovascular-related diseases and skin diseases [16–19]; however, their significance in psoriasis is uncertain.

Understanding how abnormal platelet structure and function relate to the presence and severity of psoriasis may refine our understanding of disease pathophysiology and contribute to new insights in risk stratification and prognosis. We therefore performed a systematic review and meta-analysis to determine the association of platelet indices with psoriasis.

## Patients and methods

### Study design and registration

This systematic review and meta-analysis evaluated study-level data, and was reported in compliance with the Preferred Reporting Items for Systematic reviews and Meta-Analyses (PRISMA) [20] and Meta-analysis Of Observational Studies in Epidemiology (MOOSE) [21] statements. We prospectively registered review protocol details with PROSPERO (CRD42021290920); there were no protocol deviations.

### Criteria for considering studies for this review

We included all original research studies which reported platelet parameters of PLT, MPV, PDW, PCT, IPF, and PMI grouped by patients with and without psoriasis. We defined PLT as the count of platelets as measured in × 10^9^/L, MPV as the average size of individual platelets measured in fL, PDW as the variability in platelet size measured in %, PCT as plateletcrit and a reflection of the mass of platelets measured in %, IPF as the fraction of immature platelets in blood and a marker of thrombopoietic activity measured in %, and PMI as the product of PLT and MPV and a reflection of the plaque forming capacity of platelets. We excluded non-human studies, conference abstracts and presentations, case reports and series, expert opinions, and letters to the editor.

### Search methods for identification of studies

We searched MEDLINE (Ovid), Embase (Ovid), and the Cochrane Library from inception to November 2021, and employed a comprehensive set of search terms for PLT, MPV, PDW, PCT, IPF, PMI, and psoriasis in our search strategy (Online Resource 1). We placed no limits on language or publication period. We subsequently reviewed the reference and citation lists of included studies for further potentially relevant studies. We used the online software ‘Covidence’ [22] to organise the search process.

### Study selection

Two review authors (ZL and LAP) independently screened the titles and abstracts of each search result against eligibility criteria for relevant studies. Subsequently, the same two authors independently reviewed the full texts of studies identified as possibly relevant. Disagreements at each stage were adjudicated by discuss with a third review author (VM).

### Data extraction and management

Two review authors (ZL and LAP) independently extracted data from included studies into standardised spreadsheets. We recorded the following where reported: study design; population baseline characteristics, clinical dermatological characteristics such as psoriatic arthritis prevalence, Psoriasis Area and Severity Index (PASI), disease duration; comorbidities; mean PLT, MPV, PDW, PCT, IPF, and PMI measurements and standard deviation stratified into psoriasis vs control groups; and correlation of the aforementioned platelet parameters with PASI score. Where studies contrasted average platelet measurements between psoriasis and control groups, we standardised reported data as mean and standard deviation [23].

### Assessment of methodological quality

Two review authors (ZL and LAP) independently assessed risk of bias of included studies using the Newcastle–Ottawa Quality Assessment Scale (NOS) for case–control studies [24], with discrepancies adjudicated by discussion with a third author (VM). The NOS consists of a linear ‘star’ scale ranging from 0 stars (worst) to 9 stars (best). Stars may be awarded in 8 different areas classified under 3 subscales of selection, comparability, and exposure. We graded a study with a star count of at least 7 as high quality, at least 5 as fair quality, and otherwise as poor quality [25–27].

### Statistical analysis and data synthesis

We tabulated mean differences (MD) with associated confidence intervals (CI) and correlation coefficients for each included study grouped by platelet parameter, generated summary estimates with random-effects inverse-variance modelling, and graphically depicted findings using forest plots. We performed meta-analyses where studies were study numbers were sufficient and studies were not excessively clinically heterogeneous; otherwise, we performed qualitative descriptive analyses.

We used the I^2^ statistic to approximate statistical heterogeneity for each meta-analysis, and where possible used meta-regression to investigate potential sources of heterogeneity where this was significant (clinical judgement, I^2^ statistic > 50%, and where there were approximately ten or greater studies in the analysis) through inputting pre-specified covariates into a mixed-effects model [28]. We pre-specified the following covariates for meta-regression: study characteristics including retrospective vs prospective study design, methodological quality out of nine stars as per the Newcastle–Ottawa scale, sample size, and percentage male; patient attributes including average age and body mass index; clinical dermatological features such as PASI score, duration of disease, prevalence of psoriatic arthritis within psoriasis patients; as well as patient comorbidities such as prevalence of smoking, alcohol consumption, hypertension, diabetes mellitus, cardiovascular disease, metabolic syndrome, and dyslipidaemia in both psoriasis and control populations.

Where studies were assessed as overall poor quality on the NOS, and where such studies were included in quantitative meta-analysis, we performed sensitivity analyses to assess the influence of these studies by removing them from the meta-analysis. We also performed ‘leave-one-out’ sensitivity analyses for each meta-analysis to evaluate the impact of each single included study on pooled results.

Where there were at least 10 included studies in a meta-analysis, we generated contour enhanced funnel plots to formally assess for reporting bias [29]. We used Egger’s regression test to analyse potential funnel plot asymmetry, followed by visual inspection to identify the statistical significance of regions of potentially missing studies, to evaluate the likelihood of asymmetry being due to reporting bias as opposed to other factors such as poor methodological quality leading to spuriously inflated effects in smaller studies, true heterogeneity, artefact, or chance [30, 31].

We used the R [32] statistical package ‘metafor’ [33], Review Manager (RevMan) 5.4 [34], and Stata [35] to perform all analyses and generate figures.

## Results

### Search results

**Fig. 1:**
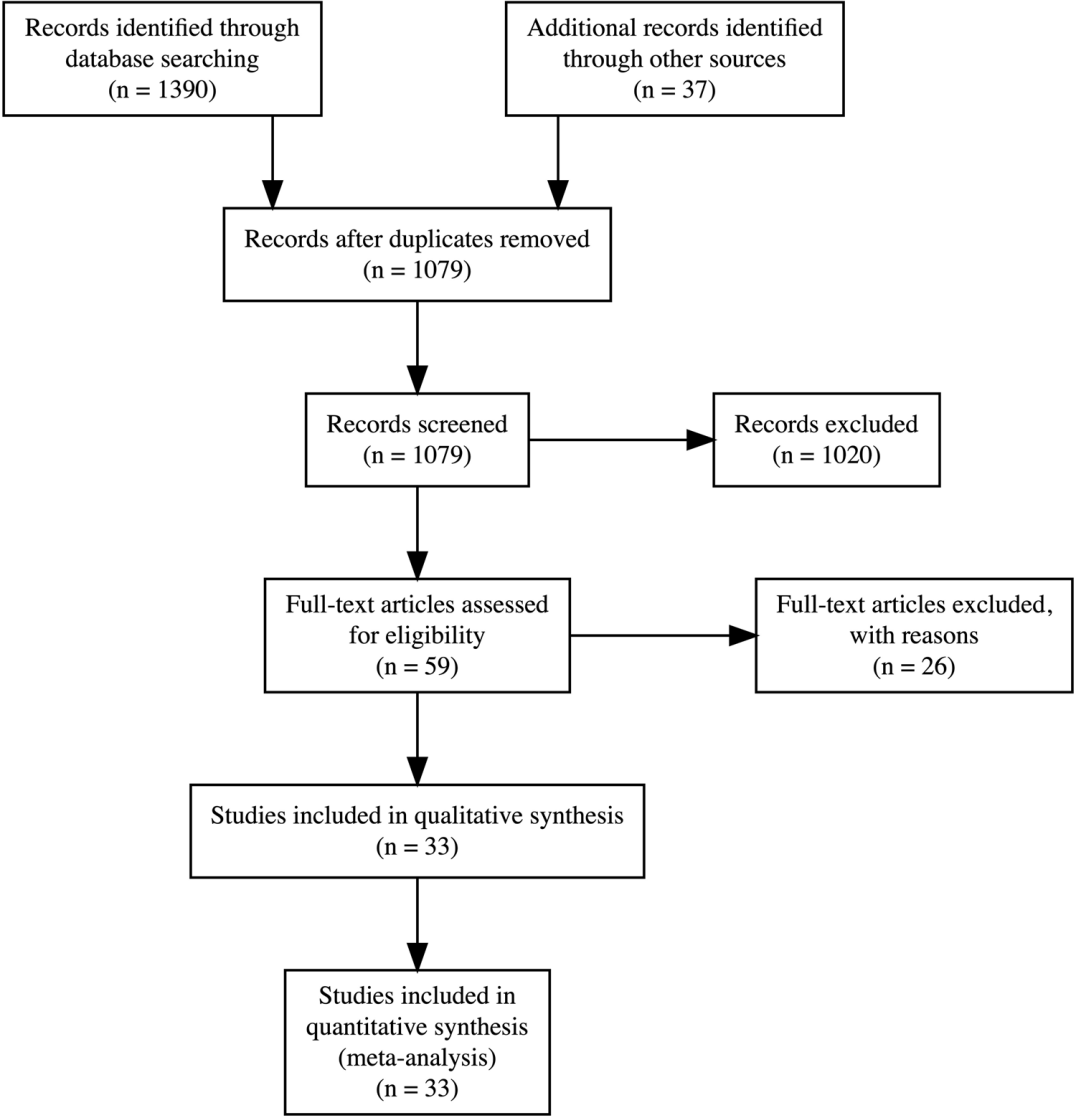
Preferred Reporting Items for Systematic Reviews and Meta-Analyses (PRISMA) flow chart. 25 full-text articles were excluded due to lack of platelet count, mean platelet volume, platelet distribution width, or plateletcrit reporting. 1 full-text article was excluded due to identical cohort analysed to included study

Our database searches returned 1,390 results, with 37 potentially relevant studies identified from other sources. After automatic deduplication, we screened the titles and abstracts of 1,079 unique studies. Fifty-nine studies underwent full-text review, from which 33 studies were included in the final review (Fig. 1).

### Description of included studies

Thirty-three studies [8, 9, 36–66] encompassing 6724 patients published between 2008 and 2021were included. All studies were case-control studies performed at a single centre. Detailed characteristics of included studies are explored in Table 1.

**Table 1:** Characteristics of included studies

Study ID	Design	Sample Size (n)	Age (mean ± SD, years)	Sex (% male)	PASI (mean ± SD)	Psoriasis Duration (mean ± SD, years)	Psoriatic Arthritis Prevalence	Diabetes Prevalence	Cardiovascular Disease Prevalence	Hypertension Prevalence	Dyslipidaemia Prevalence	Platelet Indices Reported	NOS Methodological Quality
Ahmad [36]	Retrospective	60	37.7 ± 10.3	55.0%	Not reported	Not reported	Not reported	0.0%	Not reported	0.0%	0.0%	MPV	High
Ataseven [37]	Retrospective	174	38.8 ± 14.9	37.4%	11.8 ± 7.2	Not reported	Not reported	Not reported	Not reported	Not reported	Not reported	PLT	Fair
Canpolat [8]	Prospective	201	41.0 ± 11.5	51.2%	13.6 ± 6.4	15.5 ± 3.7	23.9%	0.0%	0.0%	0.0%	0.0%	PLT, MPV	Fair
Çerman [38]	Retrospective	96	40.1 ± 14.7	56.3%	Not reported	10.3 ± 7.5	Not reported	Not reported	Not reported	Not reported	Not reported	MPV	Fair
Chandrashekar [39]	Retrospective	124	43.6 ± 12.3	83.9%	14.9 ± 9.8	1.7 ± 1.7	Not reported	0.0%	0.0%	Not reported	0.0%	MPV, PDW	High
Dincer Rota [40]	Retrospective	141	41.0 ± 23.0	44.7%	Not reported	Not reported	Not reported	6.4%	4.3%	15.6%	50.4%	PLT	High
Doğan 2017	Retrospective	272	44.0 ± 14.3	59.6%	Not reported	Not reported	Not reported	Not reported	Not reported	Not reported	Not reported	PLT, MPV	High
Erek Toprak [42]	Prospective	88	36.2 ± 13.5	46.6%	11.3 ± 7.3	Not reported	Not reported	0.0%	0.0%	0.0%	0.0%	PLT, MPV	Poor
Farag [43]	Prospective	130	40.8 ± 9.2	61.5%	12.9 ± 5.0	8.4 ± 3.4	Not reported	0.0%	0.0%	0.0%	0.0%	MPV	High
Garshick [44]	Prospective	63	43.6 ± 13.8	50.8%	4.5 ± 3.3	16.8 ± 11.1	20.0%	1.6%	Not reported	9.5%	Not reported	PLT, MPV, IPF	High
Hammad [45]	Retrospective	72	36.9 ± 6.5	61.1%	28.5 ± 18.2	12.2 ± 9.8	0.0%	Not reported	Not reported	Not reported	Not reported	PLT, MPV	Fair
Hancer [46]	Prospective	80	47.1 ± 13.7	57.5%	10.8 ± 6.4	15.3 ± 9.0	47.5%	0.0%	0.0%	0.0%	0.0%	PLT, MPV, PDW	Fair
Işik 2016	Retrospective	89	45.1 ± 14.1	50.6%	16.3 ± 11.2	13.7 ± 11.3	Not reported	Not reported	0.0%	Not reported	Not reported	MPV	Poor
Karabudak [48]	Prospective	40	22.0 ± 3.1	100.0%	13.0 ± 7.0	4.0 ± 4.0	Not reported	0.0%	0.0%	0.0%	0.0%	MPV	Fair
Kim [50]	Retrospective	277	38.8 ± 15.3	56.3%	Not reported	Not reported	Not reported	0.0%	0.0%	0.0%	0.0%	PLT, MPV, PDW	Fair
Kim [51]	Retrospective	205	36.3 ± 15.5	51.2%	Not reported	Not reported	0.0%	Not reported	Not reported	Not reported	Not reported	PLT	High
Kılıç 2017	Retrospective	131	39.7 ± 14.3	43.5%	8.4	16.5 ± 12.0	0.0%	5.3%	3.1%	7.6%	Not reported	MPV	Fair
Korkmaz [52]	Retrospective	149	43.8 ± 9.9	51.7%	2.8 ± 2.0	Not reported	0.0%	0.0%	0.0%	0.0%	Not reported	PLT, MPV, PDW	Fair
Mahrous [53]	Prospective	60	41.3 ± 9.1	71.7%	12.7 ± 4.0	7.5 ± 2.8	Not reported	0.0%	0.0%	0.0%	0.0%	MPV	Fair
Özkur 2020	Retrospective	58	44.3 ± 14.9	39.7%	5.5 ± 3.4	Not reported	Not reported	0.0%	0.0%	0.0%	0.0%	PLT, MPV	High
Pektas [55]	Retrospective	300	41.5 ± 12.3	49.7%	7.8 ± 6.9	Not reported	Not reported	0.0%	Not reported	Not reported	Not reported	PLT, MPV, PCT	Fair
Polat [56]	Retrospective	92	35.3 ± 9.2	50.0%	9.1 ± 8.8	13.2 ± 8.2	0.0%	Not reported	Not reported	Not reported	Not reported	PLT, MPV	High
Raghavan [57]	Prospective	100	48.4 ± 13.6	81.0%	15.9 ± 2.5	5.6 SD not reported	Not reported	0.0%	0.0%	0.0%	0.0%	PLT, MPV	Fair
Saleh [9]	Prospective	50	29.0 ± 9.7	50.0%	22.6 ± 18.1	6.2 ± 6.0	Not reported	0.0%	0.0%	0.0%	0.0%	MPV	High
Sharma [58]	Prospective	62	36.0 ± 12.0	35.8%	Not reported	Not reported	Not reported	4.8%	Not reported	1.6%	Not reported	PLT, MPV, PDW, PCT	Fair
Sirin [59]	Prospective	110	36.4 ± 12.2	50.9%	10.2 ± 9.6	9.1 ± 7.8	1.7%	0.0%	0.0%	0.0%	0.0%	PLT, MPV, PDW	Fair
Tamagawa-Mineoka [60]	Prospective	43	49.3 ± 12.4	69.8%	15.8 ± 1.7	Not reported	Not reported	0.0%	0.0%	0.0%	0.0%	PLT	Fair
Ünal [61]	Retrospective	520	33.7 SD not reported	51.0%	Not reported	8.7 ± 8.4	5.3%	0.0%	0.0%	0.0%	0.0%	PLT, MPV	Fair
Wang [62]	Retrospective	1431	43.5 ± 14.3	68.8%	Not reported	Not reported	5.2%	Not reported	Not reported	Not reported	Not reported	PLT	High
Yavuz [63]	Prospective	90	41.0 ± 14.3	50.0%	8.1 ± 5.2	9.4 ± 7.3	Not reported	0.0%	0.0%	0.0%	0.0%	PLT, MPV	Fair
Yorulmaz [64]	Retrospective	342	43.4 ± 14.0	59.6%	7.5 ± 7.6	9.2 ± 9.0	12.3%	Not reported	Not reported	Not reported	Not reported	PLT, PDW	High
Yurtdaş 2014	Prospective	88	38.2 ± 9.0	59.1%	16.0 ± 11.0	7.0 ± 5.0	0.0%	0.0%	0.0%	0.0%	0.0%	PLT	High
Zhou [66]	Retrospective	986	40.7 ± 16.4	54.6%	Not reported	Not reported	0.0%	0.0%	0.0%	0.0%	0.0%	PLT, MPV, PDW, PCT	Fair

*MPV* mean platelet volume, *NOS* Newcastle–Ottawa scale, *PASI* psoriasis area and severity index, *PCT* Plateletcrit, *PDW* platelet distribution width, *PLT* platelet count, *SD* standard deviation

### Methodological quality

Methodological quality was variable across studies, as assessed by the NOS. Thirteen studies [9, 36, 39–41, 43, 44, 51, 54, 56, 62, 64, 65] were regarded as being high quality, eighteen studies [8, 37, 38, 45, 46, 48–50, 52, 53, 55, 57–61, 63, 66] fair quality, and two studies [42, 47] were identified as poor quality. All studies attained 3 out of 3 stars in the exposure subscale; overall methodological quality was differentiated in the subscales of selection and comparability. Studies ranged from 1 to 4 stars in selection, with lower star ratings arising from concerns of case definition, representativeness of cases, and selection of controls. Studies ranged from 0 to 2 stars in comparability of cases and controls on the basis of design or analysis. The complete NOS assessment can be found in Online Resource 2.

### Meta-analyses of platelet parameters

Platelet count.

### Presence of psoriasis

**Fig. 2:**
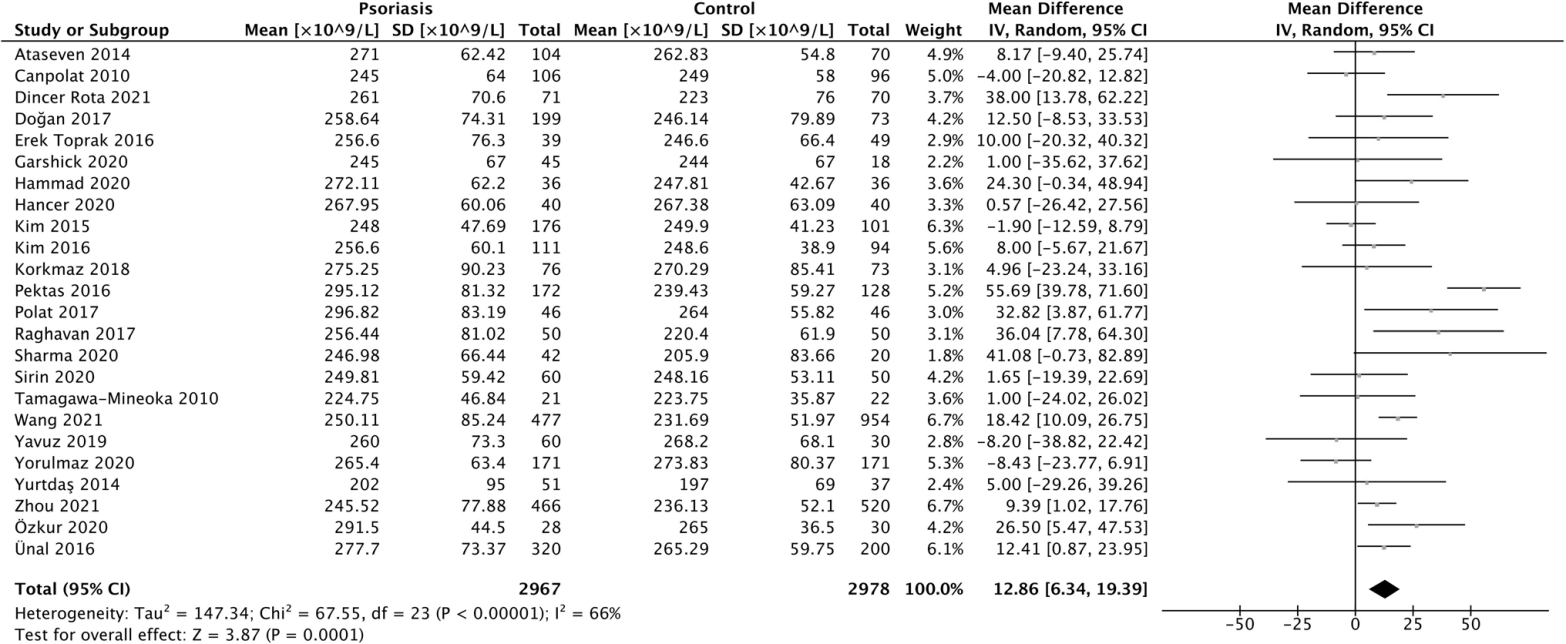
Forest plot PLT MD in patients with and without psoriasis

From 24 studies [8, 37, 40–42, 44–46, 50–52, 54–66] involving 5,944 patients, we found that patients with psoriasis had a statistically significant increased PLT compared to controls (MD 12.86 × 10^9^/L, 95% CI 6.34–19.39, *p* < 0.001) (Fig. 2). We performed a sensitivity analysis to remove one study [42] identified as being of poor methodological quality; resulting PLT differences were similar (MD 12.96 × 10^9^/L, 95% CI 6.27–19.66, *p* < 0.001). Leave-one-out sensitivity analyses were not significant (Online Resource 3).

Statistical heterogeneity was substantial (*I*^2^ statistic 66%). We used meta-regression of pre-specified covariates to investigate this further, and found that prevalence of smoking, comorbid cardiovascular disease, hypertension, and dyslipidaemia were statistically significant positive modifiers of the MD (regression coefficients 62.97, 733.86, 61.86, 165.52; p-values 0.015, 0.020, 0.022 and 0.040 respectively). In particular, we found that smoking prevalence accounted for up to 100% of observed statistical heterogeneity (Online Resource 4).

Egger’s regression test for funnel plot asymmetry using a weighted regression with multiplicative dispersion model did not reveal significant asymmetry (*p* = 0.654); this was consistent with visual inspection (Online Resource 5).

### Severity of psoriasis

**Fig. 3:**
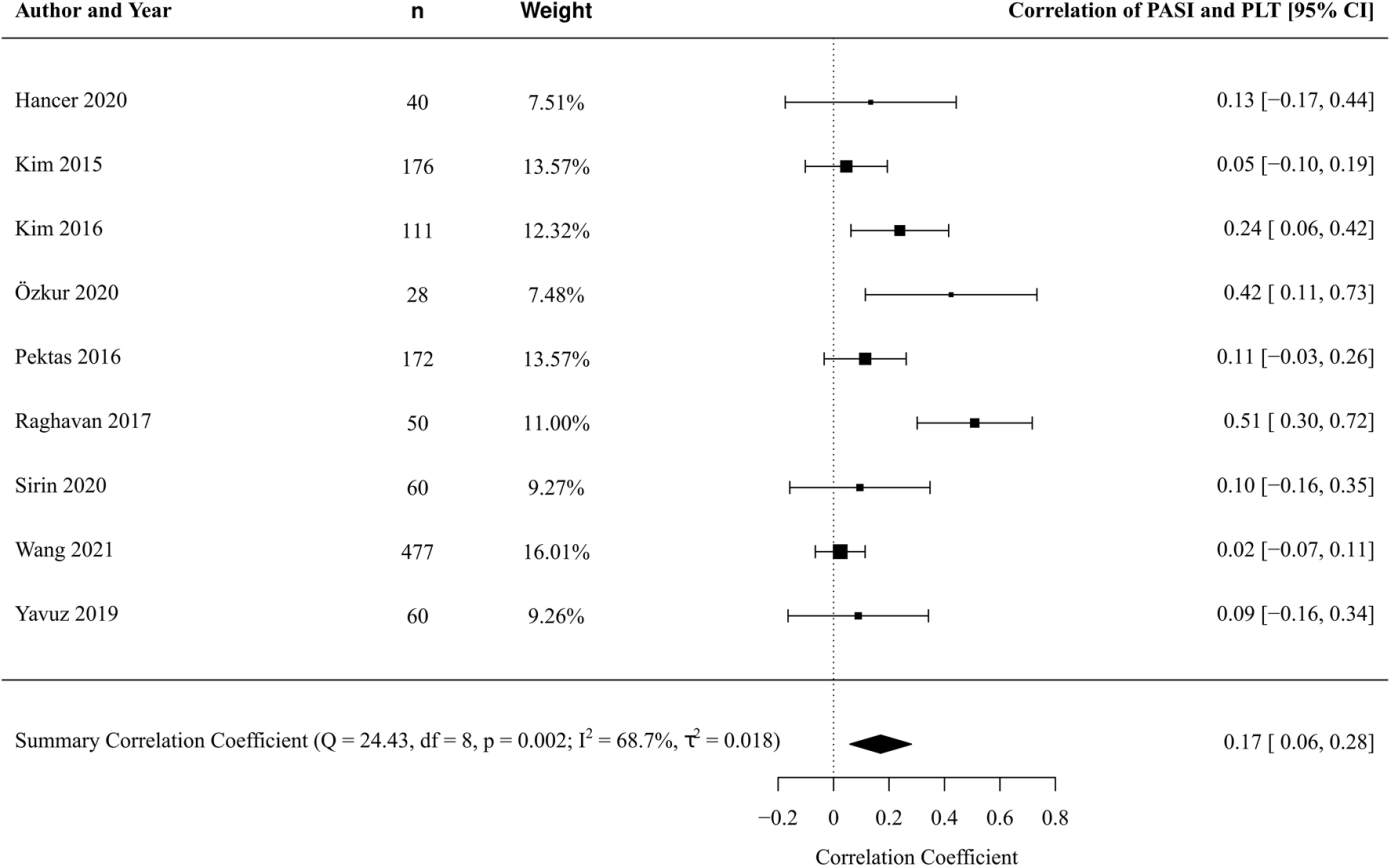
Forest plot PLT correlation with PASI in patient with psoriasis

From 9 studies [46, 50, 51, 54, 55, 57, 59, 62, 63] involving 2,731 patients, we found a weak correlation between PLT and PASI in patients with psoriasis (correlation coefficient 0.17, 95% CI 0.06–0.28, *p* = 0.003) (Fig. 3). There was a substantial degree of statistical heterogeneity (*I*^2^ statistic 69%); however, meta-regression was not possible due to insufficient study number for analysis. Similarly, formal testing for reporting bias was not possible due to low study numbers. Leave-one-out sensitivity analyses were not significant (Online Resource 6).

### Mean platelet volume

#### Presence of psoriasis

**Fig. 4:**
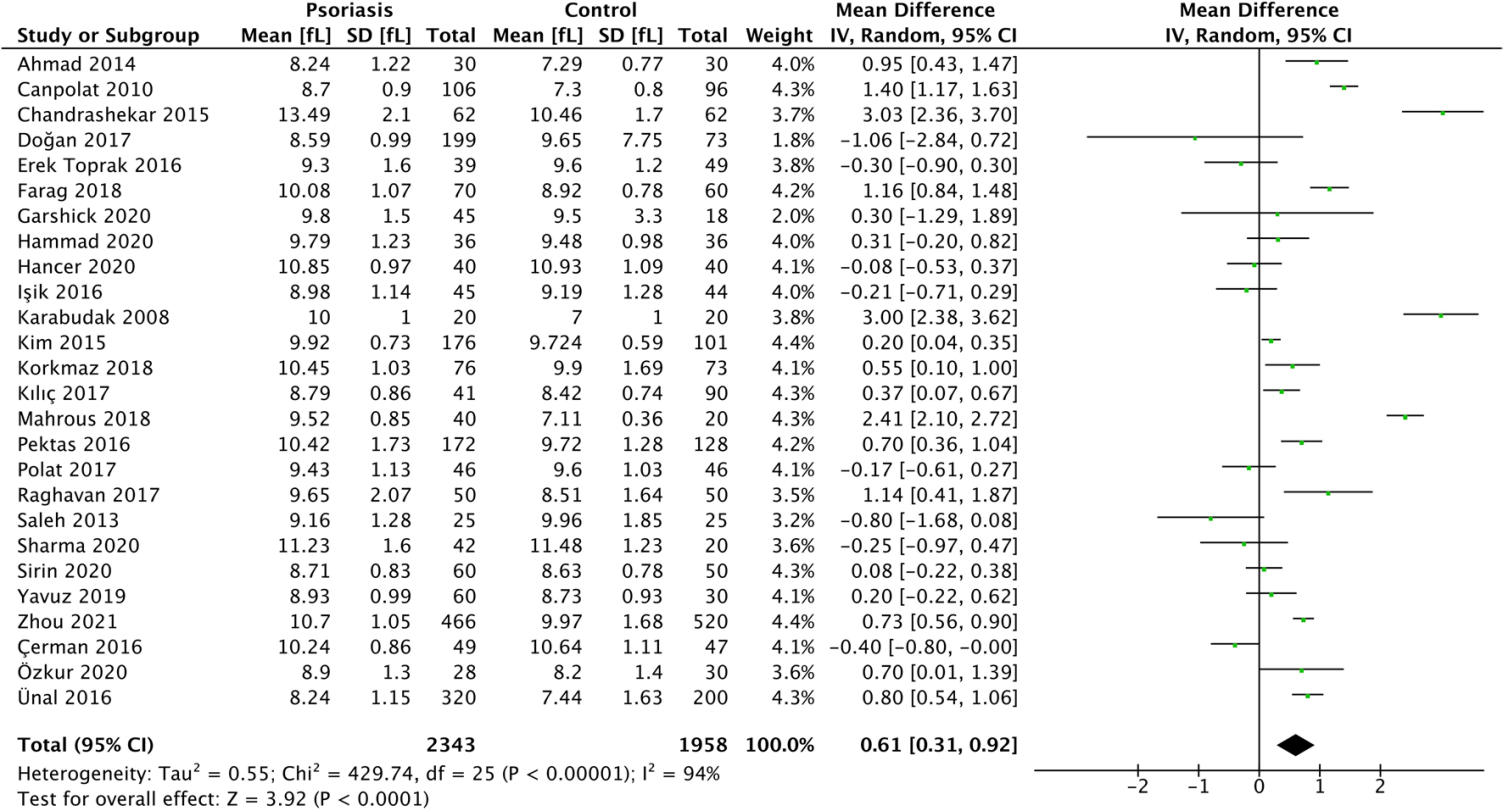
Forest plot MPV MD in patients with and without psoriasis

From 26 studies [8, 9, 36, 38, 39, 41–50, 52–59, 61, 63, 66] involving 4,300 patients, we found that patients with psoriasis had a statistically significant increased MPV compared to controls (MD 0.61 fL, 95% CI 0.31–0.92, *p* < 0.001) (Fig. 4). We performed a sensitivity analysis to remove two studies [42, 47] identified as being of poor methodological quality; resulting MPV differences were similar (MD 0.69 fL, 95% CI 0.37–1.00, *p* < 0.001). Leave-one-out sensitivity analyses were not significant (Online Resource 7).

Statistical heterogeneity was considerable (I^2^ statistic 94%). We used meta-regression of pre-specified covariates to investigate this further, and found that percentage male and duration of psoriasis were statistically significant modifiers of the MD (regression coefficients 5.33, − 0.14; *p*-values < 0.001, 0.008 respectively). These accounted for up to 60% and 29% of observed statistical heterogeneity respectively in univariable analysis; however, residual heterogeneity remained considerable (Online Resource 8).

Egger’s regression test for funnel plot asymmetry using a weighted regression with multiplicative dispersion model did not reveal significant asymmetry (*p* = 0.889); this was consistent with visual inspection (Online Resource 9).

#### Severity of psoriasis

**Fig. 5:**
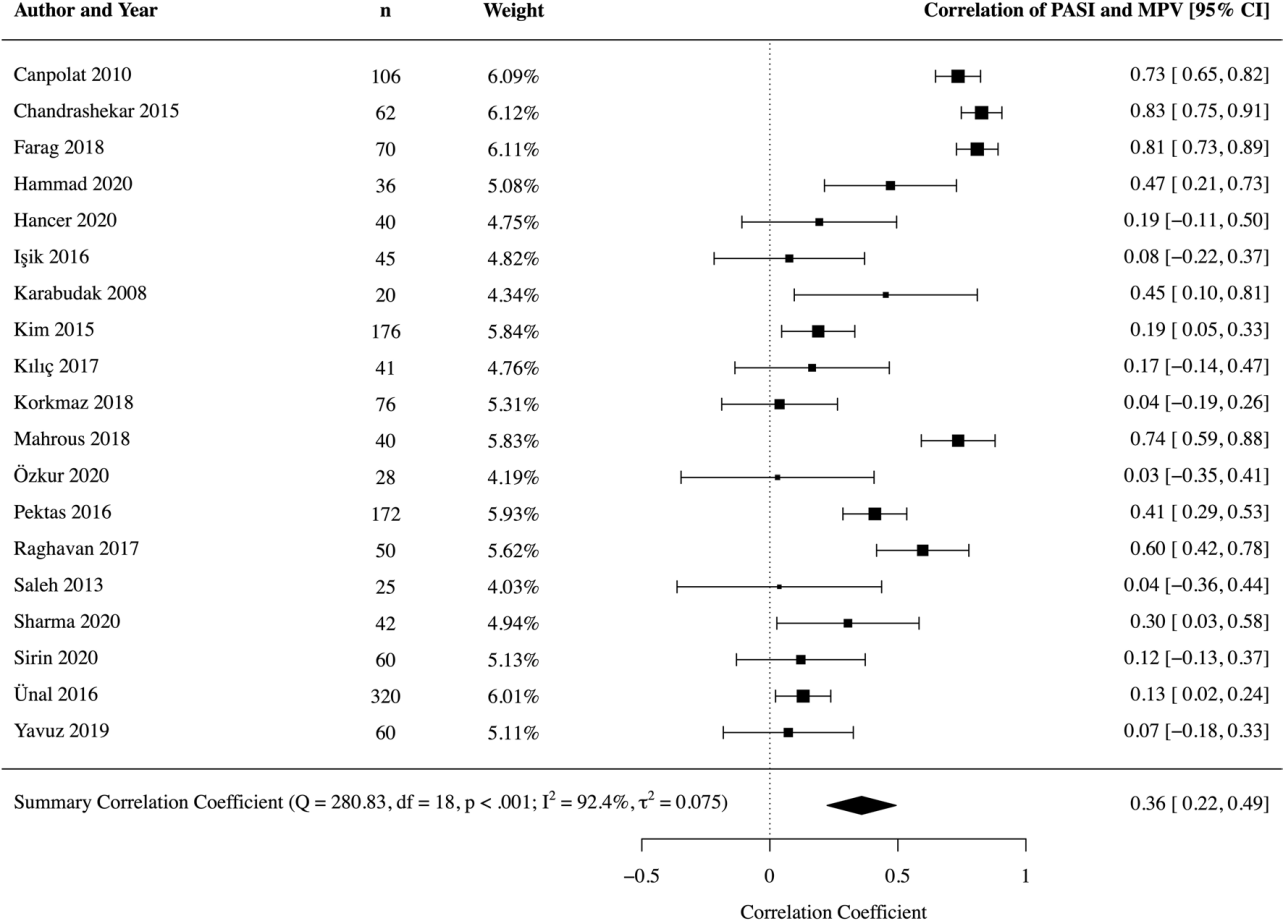
Forest plot MPV correlation with PASI in patient with psoriasis

From 19 studies [8, 9, 39, 43, 45–50, 52–55, 57–59, 61, 63] involving 2,583 patients, we found a weak correlation between MPV and PASI in patients with psoriasis (correlation coefficient 0.36, 95% CI 0.22–0.49, *p* < 0.001) (Fig. 5). We performed a sensitivity analysis to remove one study [47] identified as being of poor methodological quality; resulting correlation was similar (correlation coefficient 0.37 fL, 95% CI 0.23–0.51, *p* < 0.001). Leave-one-out sensitivity analyses were not significant (Online Resource 10).

There was a considerable degree of statistical heterogeneity (I^2^ statistic 92%). We used meta-regression of pre-specified covariates to investigate this further, and found that percentage male was a statistically significant modifier of the correlation coefficient (regression coefficient 1.12; *p*-value 0.004). This accounted for up to 33% of observed statistical heterogeneity in univariable analysis; however, residual heterogeneity remained considerable (Online Resource 11).

Egger’s regression test for funnel plot asymmetry using a weighted regression with multiplicative dispersion model suggested significant asymmetry (*p* = 0.003). Visual inspection suggested potential missing studies in the bottom right hand side of the funnel plot (Online Resource 12). Given that most of this area contains regions of high significance as represented by the shaded triangles, the underlying cause of asymmetry is more likely true heterogeneity, artefact, or chance than reporting bias, though the latter cannot be excluded.

### Platelet distribution width

#### Presence of psoriasis

**Fig. 6:**
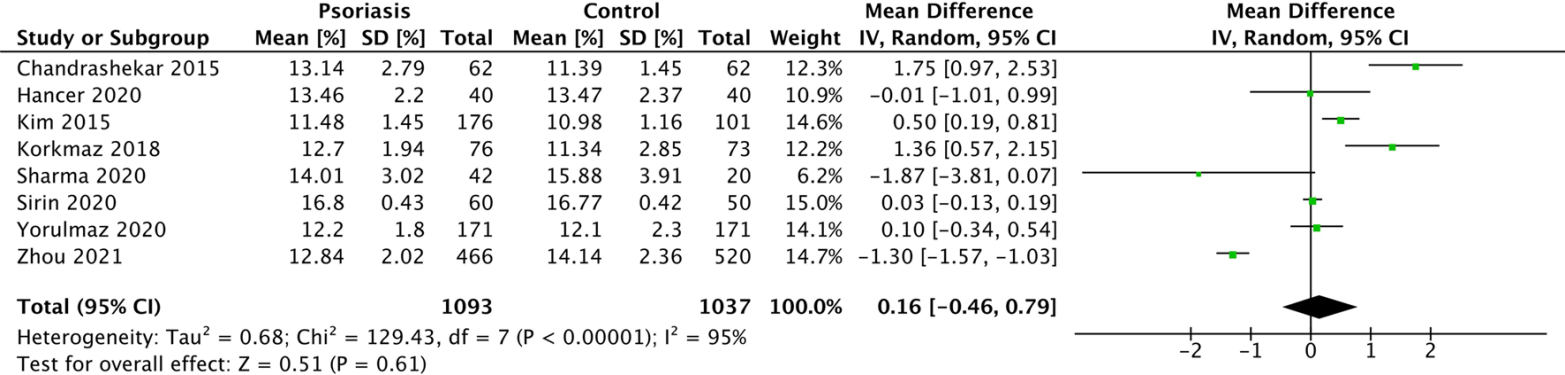
Forest plot PDW MD in patients with and without psoriasis

From 8 studies [39, 46, 50, 52, 58, 59, 64, 66] involving 2,130 patients, we found no significant difference in PDW in patients with psoriasis compared to controls (MD 0.16%, 95% CI − 0.46–0.79, *p* = 0.610) (Fig. 6). There was a substantial degree of statistical heterogeneity (*I*^2^ statistic 95%); however, meta-regression was not possible due to insufficient study number for analysis. Similarly, formal testing for reporting bias was not possible due to low study numbers. Leave-one-out sensitivity analyses were not significant (Online Resource 13).

#### Severity of psoriasis

**Fig. 7:**
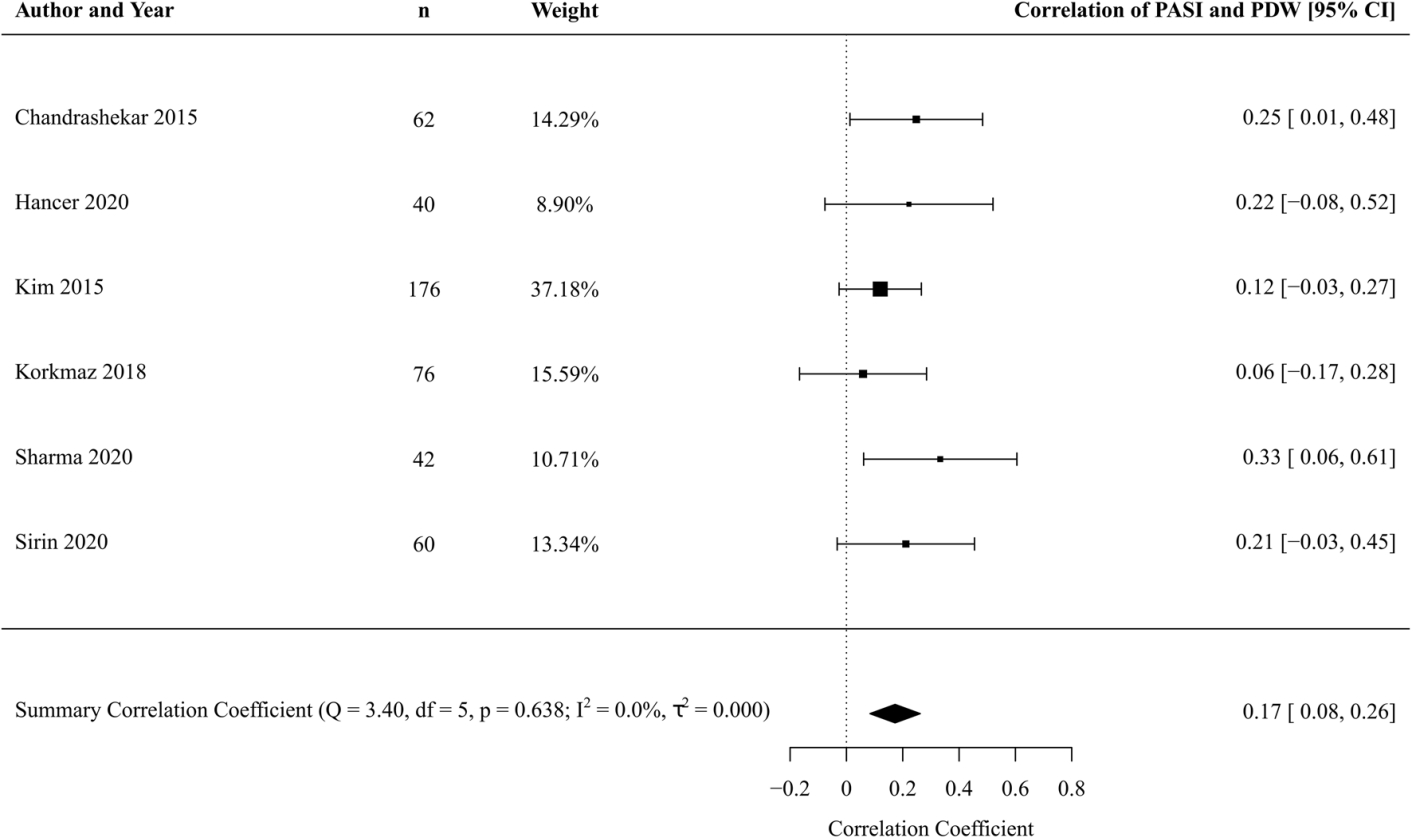
Forest plot PDW correlation with PASI in patient with psoriasis

From 6 studies [39, 46, 50, 52, 58, 59] involving 802 patients, we found a weak correlation between PDW and PASI in patients with psoriasis (correlation coefficient 0.17, 95% CI 0.08–0.26, *p* < 0.001) (Fig. 7). Statistical heterogeneity was absent (*I*^2^ statistic 0%). Formal testing for reporting bias was not possible due to low study numbers. Leave-one-out sensitivity analyses were not significant (Online Resource 14).

### Plateletcrit

#### Presence of psoriasis

**Fig. 8:**
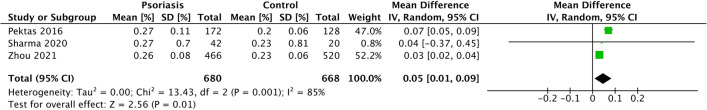
Forest plot PCT MD in patients with and without psoriasis

From 3 studies [55, 58, 66] involving 1,348 patients, we found that patients with psoriasis had a statistically significant increased PCT compared to controls (MD 0.05%, 95% CI 0.01–0.09, *p* = 0.010) (Fig. 8). There was a substantial degree of statistical heterogeneity (I^2^ statistic 85%); however, meta-regression was not possible due to insufficient study number for analysis. Similarly, formal testing for reporting bias was not possible due to low study numbers.

#### Severity of psoriasis

One study [55] involving 300 patients reported a weak correlation between PCT and PASI in patients with psoriasis (correlation coefficient 0.11, 95% CI 0.00–0.22, *p* = 0.001).

### Immature platelet fraction

#### Presence of psoriasis

One study [44] involving 63 patients reported no significant difference in IPF in patients with psoriasis compared to controls (MD −0.20%, 95% CI − 1.89–1.49, *p* = 0.890).

#### Severity of psoriasis

No studies reported on the correlation of IPF and PASI in patients with psoriasis.

### Platelet mass index

#### Presence of psoriasis

One study [61] involving 520 patients reported a significant elevation in PMI in patients with psoriasis compared to controls (MD 295, 95% CI 190–400, *p* < 0.001).

#### Severity of psoriasis

One study [61] involving 520 patients did not find a significant correlation of PMI and PASI in patients with psoriasis (correlation coefficient − 0.02, 95% CI -0.13–0.09, *p* = 0.69).

## Discussion

This systematic review and meta-analysis found that patients with psoriasis have elevated PLT, MPV, and PCT, but not PDW, measurements compared to controls. Moreover, PLT, MPV, and PDW were weakly correlated with PASI in patients with psoriasis. Further studies are needed to clarify the relationship between IPF and PMI and psoriasis, as well as the correlation between PDW, IPF, and PMI with PASI.

Included studies displayed variable methodological quality as assessed by NOS; however, sensitivity analyses removing two studies deemed to be poor quality and at high risk of bias showed robustness of our findings, and meta-regression of NOS methodological quality out of nine stars did not significantly modify effect size where it could be performed. Heterogeneity of included studies was considerable in many meta-analyses. Our meta-regression revealed cardiovascular disease and its risk factors of smoking, diabetes, and dyslipidaemia to be significant modifiers of the PLT mean difference in those with and without psoriasis, percentage male and duration of psoriasis to be significant modifiers of the MPV mean difference in those with and without psoriasis, and percentage male to be a significant modifier of the correlation coefficient between MPV and PASI. Future high-quality studies controlling for the aforementioned factors are needed to clarify the independence of our results against these potential confounding influences. Egger’s regression test for funnel plot asymmetry revealed statistical asymmetry in the analysis of correlation between MPV and PASI; however, visual inspection of the contour enhanced funnel plot showed potentially missing studies, if any, isolated to high significance regions on the plot. This suggested an increased likelihood of clinical and statistical heterogeneity between included studies as the underlying cause of funnel plot asymmetry, rather than reporting bias.

Our study extends the findings of a recent meta-analysis by Li et al. (2021) of 22 studies including 3287 patients which found that higher MPV and PDW, but not PLT, were associated with presence of psoriasis [67]. The discrepancies between PLT and PDW conclusions between studies is likely attributable to differences in number of included studies; indeed, our meta-analyses were able to include all 22 studies reported by Li et al. as well as an additional 11 relevant studies to investigate platelet indices in both presence and severity of psoriasis. Moreover, our study was able to review the state of evidence for other platelet parameters including PCT, IPF, and PMI, and found that PCT was elevated in patients with psoriasis.

Beyond their primary haemostatic role, platelets have been found to contribute to diverse immune regulatory processes. These include the expression of surface immune-related receptors such as P-selectin and CD40 ligand which facilitate leukocyte recruitment to sites of cutaneous endothelial inflammation, as well as the release of chemokines, pro-inflammatory factors, and cytokine-like molecules into the circulation upon activation [68, 69]. Studies suggest that platelet activation occurs in patients with inflammatory cutaneous disease such as psoriasis, urticaria, atopic dermatitis [70]. Berrettini et al. found that patients with psoriasis had a significantly higher plasma level of platelet chemokine β-thromboglobulin and incidence of spontaneous platelet hyperaggregability compared with control subjects [71]. Tamagawa-Mineoka et al. reported increased plasma levels of β-thromboglobulin and platelet factor 4 (another platelet chemokine) in patients with psoriasis which were both correlated with PASI and significantly reduced following successful treatment, suggesting a pathomechanistic contribution to disease activity [72]. It makes sense then that platelet function indices may be associated with psoriasis. MPV measures platelet size which increases with platelet activation [73]; as an acute phase reactant like C-reactive protein which can similarly be elevated in psoriasis [74], increases in PLT may be reflective of the underlying psoriatic inflammatory milieu; as a measure of the mass of platelets and proportional to both PLT and MPV, an increased PCT follows.

As a measure of variability in platelet size and shape, mounting evidence suggests that PDW may be a more specific marker of platelet activation than MPV, due to the former’s elevation in platelet anisocytosis after pseudopodia formation in platelet activation, but not in platelet distension following simple platelet swelling [19, 75]. While our study was able to demonstrate a relationship for MPV but not PDW, the interpretation of this finding is confounded by the disparate evidence base for the two biomarkers (26 vs 8 studies, respectively) and considerable between-study heterogeneity. Future studies are needed to validate the role of PDW in psoriasis.

Our results should be considered with the following limitations. Although we were able to perform meta-regressions to explore the effects of 13 pre-specified covariates on effect measures, and were able to identify significant effect modifiers, residual heterogeneity remained in all but the PLT meta-analysis. This persisting heterogeneity may be attributable to systemic differences in unreported study population or clinical factors. Additionally, low study numbers in some analyses meant that even though meta-analysis could be performed, both meta-regression assessment of heterogeneity and funnel plot assessment of reporting bias could not be performed, despite considerable heterogeneity. All included studies were single centre; large, high quality multicentre studies could improve external validity. Finally, the inconsistent reporting of treatment regimens used by patients with psoriasis meant that we were unable to account for the influence of specific immunosuppressive regimens (e.g. topical therapy vs phototherapy vs oral immunosuppressives vs biologics) on platelet indices.

This study identifies multiple opportunities for future research. Cheap and accessible haematological biomarkers of psoriasis presence and severity are potentially useful adjuncts to the purely clinical scoring systems in use currently—the PASI and the Psoriasis Global Assessment (PGA) scores—and indeed may be incorporated into such systems to further refine their predictive potential [76]. Whether or not platelet indices could contribute to disease diagnosis in clinically or pathologically dubious cases, or be prognostic for future adverse outcomes such as the development of psoriatic arthritis, cardiovascular disease, major adverse cardiac events, or mortality remains poorly understood. For platelet indices to be easily implementable in the clinical context, cut-off values distinguishing between normality and elevation should be developed and validated. Lastly, while the scope of this review was limited to specific platelet indices, other full blood examination biomarkers such as the neutrophil–lymphocyte ratio, platelet-lymphocyte ratio, and red cell distribution width have been found to be disturbed in psoriasis and systemic conditions associated with inflammation [10, 77–81]. The incorporation of clinical, pathological, and haematological biomarkers into validated predictive models could enhance all aspects of disease management and patient care.

This systematic review and meta-analysis found that PLT, MPV, and PCT are significantly elevated in patients with psoriasis compared to controls, and that PLT, MPV, and PDW are weakly correlated with PASI. Future studies are needed to evaluate the independent diagnostic and prognostic potentials of these biomarkers in patients with psoriasis.

## Supplementary Information

Below is the link to the electronic supplementary material.

Supplementary file1 (PDF 954 kb)
