# Supplementary material for: The association between platelet indices and presence and severity of psoriasis: a systematic review and meta-analysis
Source: Clin Exp Med. 2022 Apr 4;23(2):333–46. doi: 10.1007/s10238-022-00820-5 (PMC10224874; doi:10.1007/s10238-022-00820-5)
Supplement: Supplementary file 1 — Supplementary file1 (PDF 954 kb) [file 10238_2022_820_MOESM1_ESM.pdf]

The Association Between Platelet Indices and Presence and Severity of Psoriasis – A Systematic Review and Meta-Analysis

Clinical and Experimental Medicine

Z Liu[1-4], BBiomed, MD(Distinct) (Corresponding Author), https://orcid.org/0000-0002-6114-8629
LA Perry[3,4], BSc, MBBS(Hons), http://orcid.org/0000-0002-7494-8406
V Morgan[1], MBBS(Hons), FACD

[1] Department of Dermatology, The Royal Melbourne Hospital, Parkville, Australia
[2] Department of Medicine, Melbourne Medical School, Faculty of Medicine, Dentistry and Health Sciences, The University of Melbourne, Parkville, Australia
[3] Department of Anaesthesia, The Royal Melbourne Hospital, Parkville, Australia
[4] Department of Critical Care, Melbourne Medical School, Faculty of Medicine, Dentistry and Health Sciences, The University of Melbourne, Parkville, Australia

Corresponding author: Zhengyang Liu, zhengyang.liu.research@gmail.com

Supplementary Information

Table of Contents

Online Resource 1: Search Strategy *3*

MEDLINE (Ovid) 3

Embase (Ovid) 3

Cochrane Library 3

Online Resource 2: Newcastle-Ottawa Scale (NOS) Assessment of Methodological Quality *4*

Leave-one-out Sensitivity Analyses *5*

Online Resource 3 5

Online Resource 6 6

Online Resource 7 7

Online Resource 10 8

Online Resource 13 9

Online Resource 14 9

Meta-regression Tables *10*

Online Resource 4: Meta-regression results of PLT and psoriasis 10

Online Resource 8: Meta-regression results of MPV and psoriasis 11

Online Resource 11: Meta-regression results of MPV and PASI 12

Supplemental Figures *13*

Online Resource 5 13

Online Resource 9 14

Online Resource 12 15

Online Resource 1: Search Strategy

MEDLINE (Ovid)

1. exp Mean Platelet Volume/ or mean platelet volume.mp. or MPV.mp.
2. exp Platelet Count/ or (platelet$ or platelet count$ or platelet number$).mp.
3. (platelet distribution width$ or PDW).mp.
4. (plateletcrit$ or PCT).mp.
5. exp Cell-Derived Microparticles/ or Platelet-derived microparticle$.mp.
6. (Immature platelet fraction$ or IPF).mp.
7. (platelet mass ind$ or PMI).mp.
8. psoriasis.mp. or exp Psoriasis/
9. 1 or 2 or 3 or 4 or 5 or 6 or 7
10. 8 and 9

Embase (Ovid)

1. exp Mean Platelet Volume/ or mean platelet volume.mp. or MPV.mp.
2. exp Platelet Count/ or (platelet$ or platelet count$ or platelet number$).mp.
3. (platelet distribution width$ or PDW).mp.
4. (plateletcrit$ or PCT).mp.
5. Platelet-derived microparticle$.mp. or exp platelet microparticle/
6. (Immature platelet fraction$ or IPF).mp.
7. (platelet mass ind$ or PMI).mp.
8. psoriasis.mp. or exp Psoriasis/
9. 1 or 2 or 3 or 4 or 5 or 6 or 7
10. 8 and 9
11. 10 not Conference Abstract.pt.

Cochrane Library

#1	#1 MeSH descriptor: [Mean Platelet Volume] explode all trees
#2	mean platelet volume
#3	MPV
#4	MeSH descriptor: [Platelet Count] explode all trees
#5	platelet* or platelet count* or platelet number*
#6	platelet distribution width* or plateletcrit*
#7	PDW or PCT
#8	Platelet derived microparticle*
#9	Immature platelet fraction*
#10	IPF
#11	Platelet mass ind*
#12	PMI
#13	#1 or #2 or #3 or #4 or #5 or #6 or #7 or #8 or #9 or #10 or #11 or #12
#14	Psoriasis
#15	MeSH descriptor: [Psoriasis] explode all trees
#16	#14 or #15
#17	#13 and #16

Online Resource 2: Newcastle-Ottawa Scale (NOS) Assessment of Methodological Quality

Newcastle-Ottawa Scale for Case-Control Studies	OVERALL ASSESSMENT		Selection				Comparability	Exposure		
Study			Is the case definition adequate?	Representativeness of the cases	Selection of controls	Definition of controls	Comparability of cases and controls on the basis of the design or analysis	Ascertainment of exposure	Same method of ascertainment for cases and controls	Non-response rate
	We considered astudy which scored ≥ 7 as high-quality, ≥ 5 as fair quality, and the remaining as poor quality.	Star Count	a) Requires some independent validation (e.g. >1 person/record/time/process to extract information, or reference to primary record source such as x-rays or medical/hospital records)*<br>b) Record linkage (e.g. ICD codes in database) or self-report with no reference to primary record<br>c) No description	a) All eligible cases with outcome of interest over a defined period of time, all cases in a defined catchment area, all cases in a defined hospital or clinic, group of hospitals, health maintenance organisation, or an appropriate sample of those cases (e.g. random sample)*<br>b) Not satisfying requirements in part (a), or not stated.	This item assesses whether the control series used in the study is derived from the same population as the cases and essentially would have been cases had the outcome been present.<br>a) Community controls (i.e. same community as cases and would be cases if had outcome)*<br>b) Hospital (including non-cosmetic outpatient dermatology) controls, within same community as cases (i.e. not another city) but derived from a hospitalised (including non-cosmetic outpatient dermatology) population<br>c) No description	a) If cases are first occurrence of outcome, then it must explicitly state that controls have no history of this outcome. If cases have new (not necessarily first) occurrence of outcome, then controls with previous occurrences of outcome of interest should not be excluded.*<br>b) No mention of history of outcome	A maximum of 2 stars can be allotted in this category<br>Either cases and controls must be matched in the design and/or confounders must be adjusted for in the analysis. Statements of no differences between groups or that differences were not statistically significant are not sufficient for establishing comparability. Note: If the odds ratio for the exposure of interest is adjusted for the confounders listed, then the groups will be considered to be comparable on each variable used in the adjustment. There may be multiple ratings for this item for different categories of exposure (e.g. ever vs. never, current vs. previous or never)<br>a) study controls for age *<br>b) study controls for gender *	a) secure record (eg surgical records) *<br>b) structured interview where blind to case/control status *<br>c) interview not blinded to case/control status<br>d) written self report or medical record only<br>e) no description	a) Yes*<br>b) No	a) Same for both groups*<br>b) Non-respondents described<br>c) Rate different and no designation
Ahmad 2014	High	7	0	1	0	1	2	1	1	1
Ataseven 2014	Fair	5	1	0	0	1	0	1	1	1
Canpolat 2010	Fair	6	0	0	0	1	2	1	1	1
Çerman 2016	Fair	5	1	0	0	1	0	1	1	1
Chandrashekar 2015	High	7	0	0	1	1	2	1	1	1
Dincer Rota 2021	High	7	1	0	0	1	2	1	1	1
Doğan 2017	High	7	1	0	0	1	2	1	1	1
Erek Toprak 2016	Poor	4	0	0	0	1	0	1	1	1
Farag 2018	High	7	0	0	1	1	2	1	1	1
Garshick 2020	High	9	1	1	1	1	2	1	1	1
Hammad 2020	Fair	6	0	0	0	1	2	1	1	1
Hancer 2020	Fair	6	0	0	0	1	2	1	1	1
Işik 2016	Poor	4	0	0	0	1	0	1	1	1
Karabudak 2008	Fair	6	0	0	1	1	1	1	1	1
Kim 2015	Fair	5	1	0	0	1	0	1	1	1
Kim 2016	High	8	1	0	1	1	2	1	1	1
Kılıç 2017	Fair	6	1	0	1	1	0	1	1	1
Korkmaz 2018	Fair	6	1	0	1	1	0	1	1	1
Mahrous 2018	Fair	5	0	0	0	1	1	1	1	1
Özkur 2020	High	7	0	0	1	1	2	1	1	1
Pektas 2016	Fair	5	0	0	1	1	0	1	1	1
Polat 2017	High	8	1	0	1	1	2	1	1	1
Raghavan 2017	Fair	5	0	0	0	1	1	1	1	1
Saleh 2013	High	7	0	0	1	1	2	1	1	1
Sharma 2020	Fair	5	0	0	0	1	1	1	1	1
Sirin 2020	Fair	6	0	0	0	1	2	1	1	1
Tamagawa-Mineoka 2010	Fair	6	0	0	0	1	2	1	1	1
Ünal 2016	Fair	5	1	0	0	1	0	1	1	1
Wang 2021	High	8	1	0	1	1	2	1	1	1
Yavuz 2019	Fair	5	0	0	1	1	0	1	1	1
Yorulmaz 2020	High	8	1	0	1	1	2	1	1	1
Yurtdaş 2014	High	7	0	1	0	1	2	1	1	1
Zhou 2021	Fair	6	1	0	1	1	0	1	1	1

Leave-one-out Sensitivity Analyses

Online Resource 3

Omitted study	Mean diff. with 95% CI	p-value
Ataseven 2014	13.25 [6.12, 20.39]	0.000
Canpolat 2010	13.87 [6.92, 20.81]	0.000
Dincer Rota 2021	11.99 [5.21, 18.77]	0.001
Doğan 2017	13.03 [5.91, 20.16]	0.000
Erek Toprak 2016	13.10 [6.07, 20.12]	0.000
Garshick 2020	13.28 [6.33, 20.24]	0.000
Hammad 2020	12.56 [5.55, 19.57]	0.000
Hancer 2020	13.43 [6.43, 20.43]	0.000
Kim 2015	13.93 [6.95, 20.92]	0.000
Kim 2016	13.30 [6.12, 20.48]	0.000
Korkmaz 2018	13.27 [6.25, 20.29]	0.000
Özkur 2020	12.39 [5.39, 19.39]	0.001
Pektas 2016	10.23 [5.08, 15.38]	0.000
Polat 2017	12.35 [5.45, 19.25]	0.000
Raghavan 2017	12.22 [5.36, 19.07]	0.000
Sharma 2020	12.36 [5.52, 19.20]	0.000
Sirin 2020	13.50 [6.46, 20.54]	0.000
Tamagawa-Mineoka 2010	13.46 [6.44, 20.47]	0.000
Ünal 2015	13.05 [5.83, 20.28]	0.000
Wang 2021	12.64 [5.41, 19.87]	0.001
Yavuz 2019	13.60 [6.70, 20.51]	0.000
Yorulmaz 2020	14.15 [7.37, 20.92]	0.000
Yurtdaş 2014	13.20 [6.23, 20.16]	0.000
Zhou 2021	13.26 [6.01, 20.52]	0.000

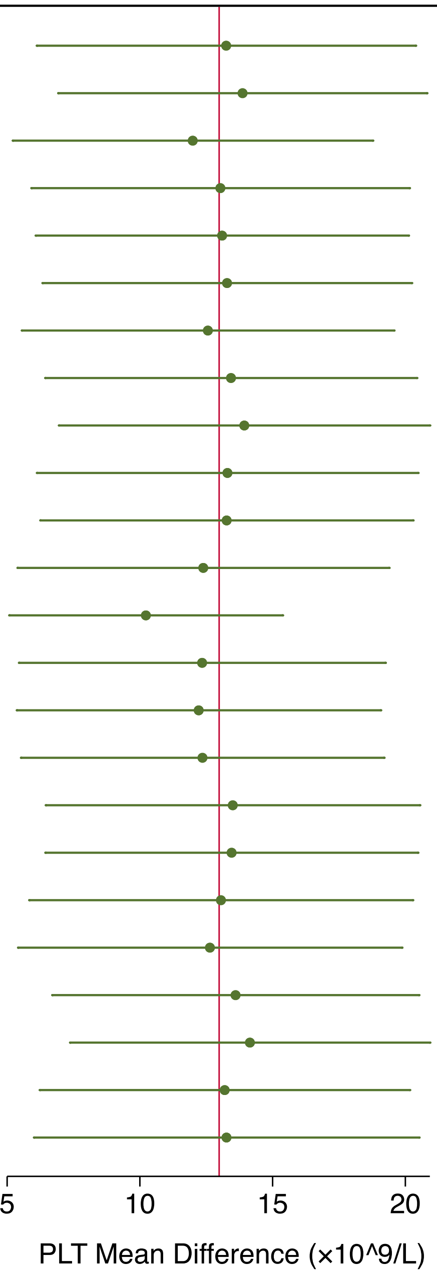

Random-effects REML model

Online resource 3: Leave-one-out sensitivity analysis for PLT and psoriasis presence.

Online Resource 6

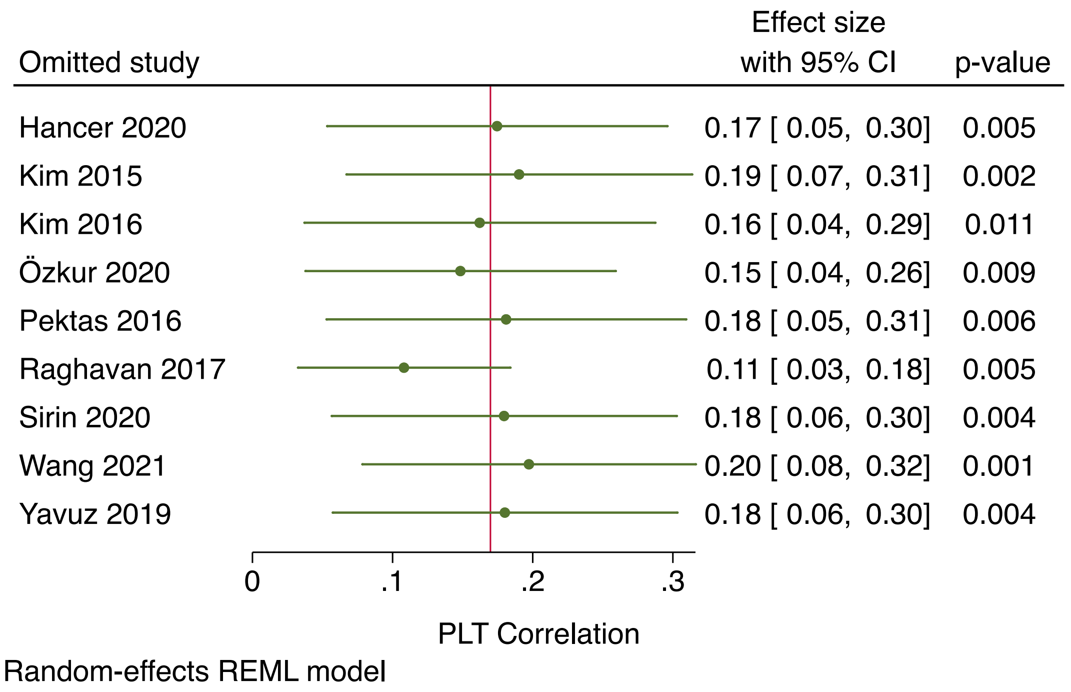

Online resource 6: Leave-one-out sensitivity analysis for PLT and psoriasis severity.

Online Resource 7

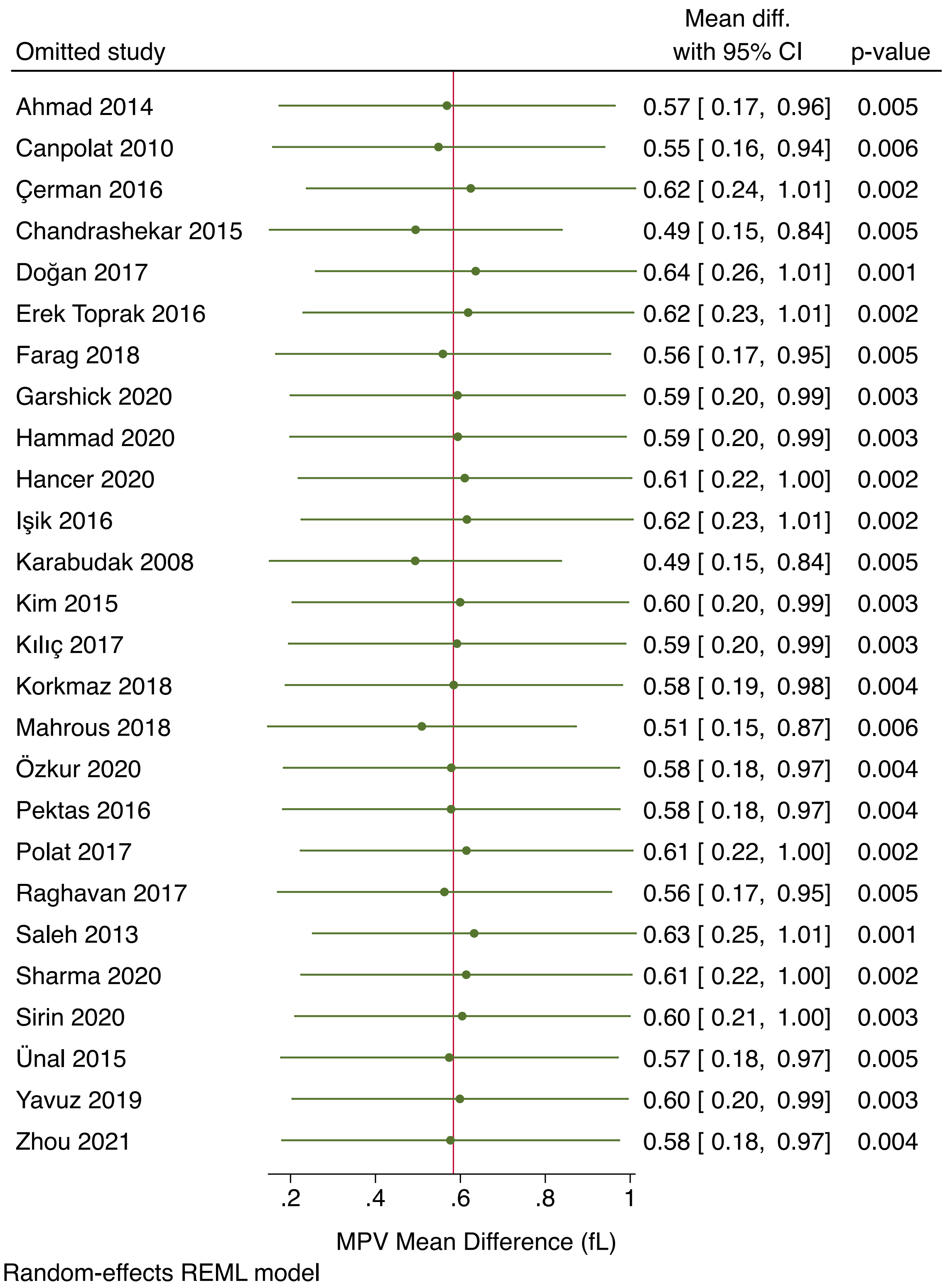

Online resource 7: Leave-one-out sensitivity analysis for MPV and psoriasis presence.

Online Resource 10

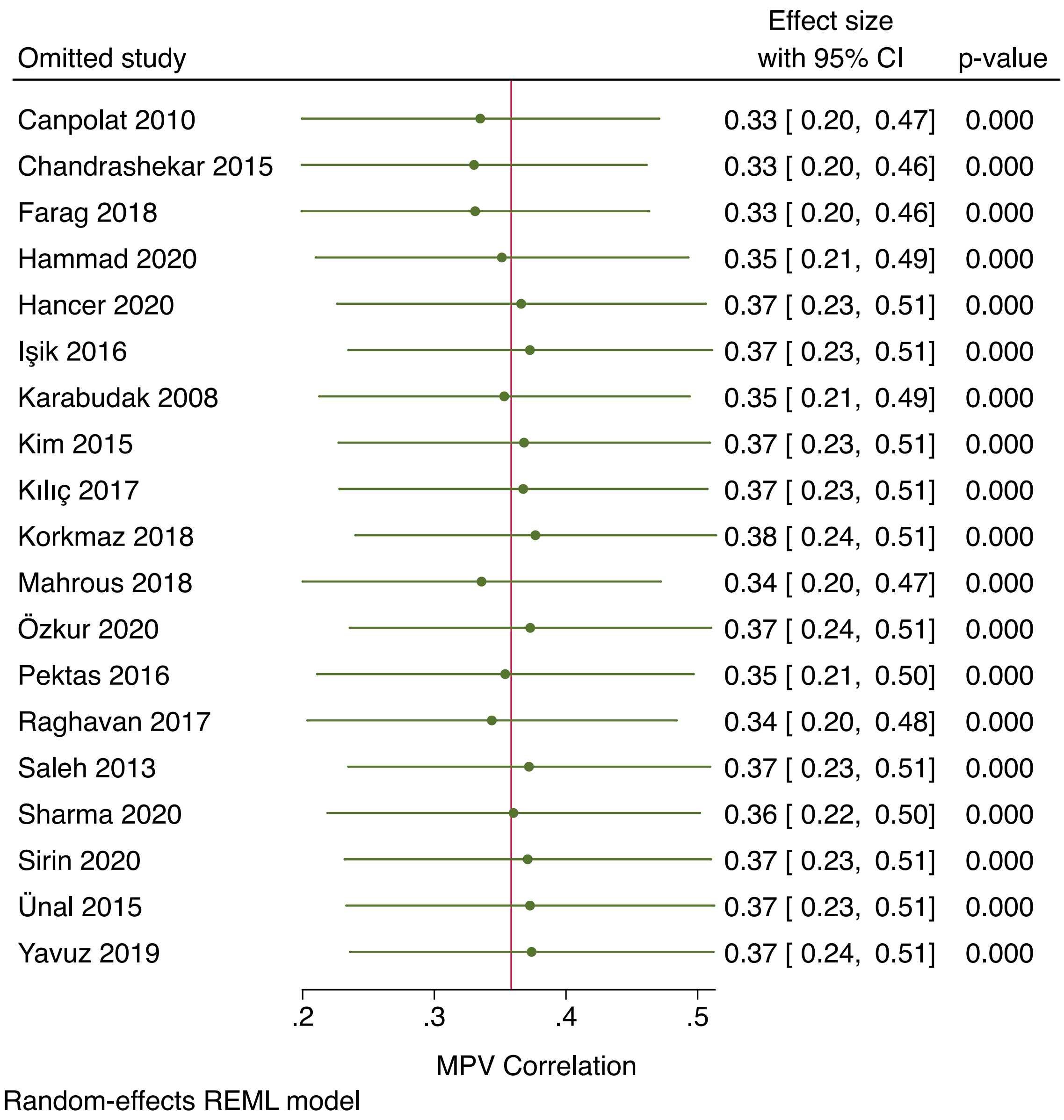

Online resource 10: Leave-one-out sensitivity analysis for MPV and psoriasis severity.

Online Resource 13

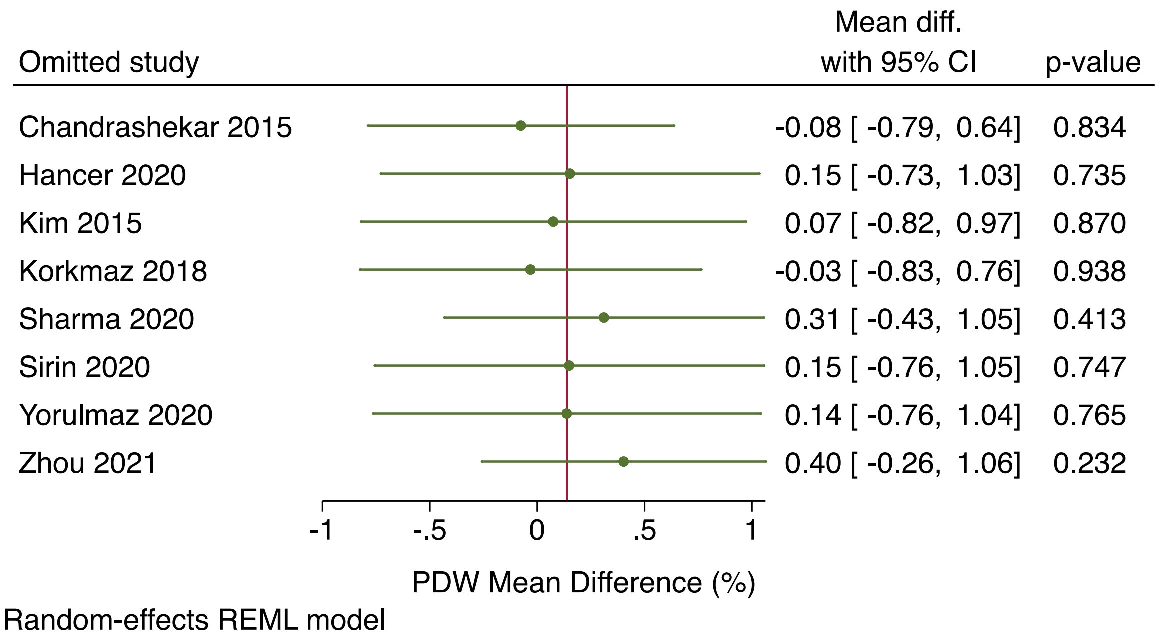

Online resource 13: Leave-one-out sensitivity analysis for PDW and psoriasis presence.

Online Resource 14

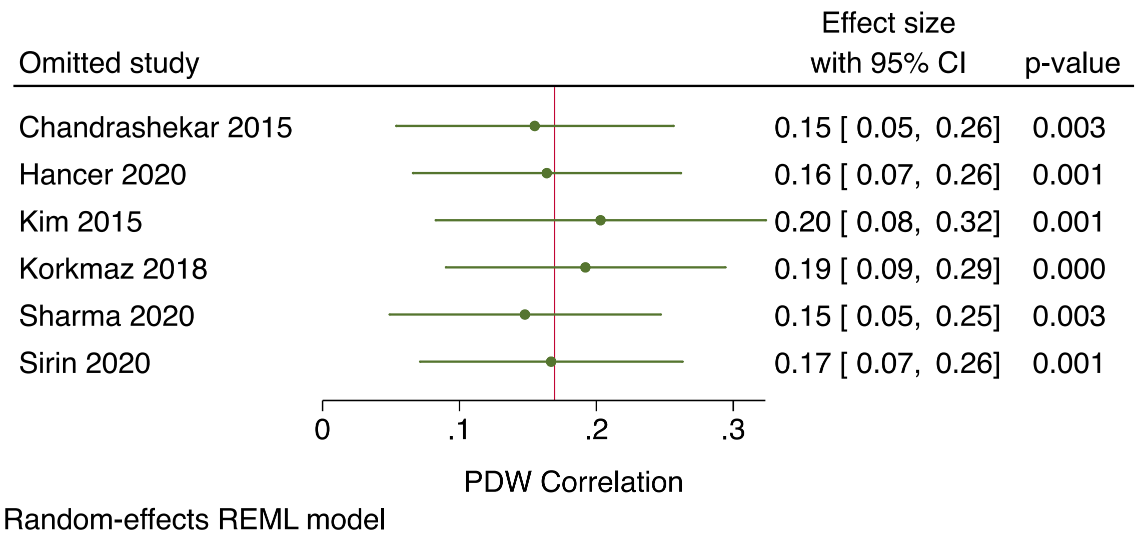

Online resource 14: Leave-one-out sensitivity analysis for PDW and psoriasis severity.

Meta-regression Tables

Online Resource 4: Meta-regression results of PLT and psoriasis

Covariate	k	Regression coefficient – magnitude of effect size modification	p-value	95% CI (lb, ub)	Heterogeneity accounted for	Residual Heterogeneity (I^2)
Smoking Prevalence	10	62.9691	0.0150	12.2312 113.7069	100.00%	0.00%
Cardiovascular Disease Prevalence	14	733.8575	0.0199	116.1744 1351.5406	61.83%	19.19%
Dyslipidaemia Prevalence	13	61.8588	0.0219	8.9671 114.7505	61.23%	23.19%
Hypertension Prevalence	16	165.5206	0.0403	7.3242 323.7169	57.09%	19.57%
Psoriatic Arthritis Prevalence	13	-45.0166	0.0868	-96.5309 6.4976	35.61%	26.97%
Diabetes Prevalence	17	442.3163	0.1045	-91.7623 976.3949	9.63%	71.20%
Study Design: Prospective vs retrospective (reference)	24	9.4656	0.2012	-5.0485 23.9797	3.81%	68.70%
Duration of Psoriasis	11	-0.9929	0.4827	-3.7651 1.7792	0.00%	43.99%
Percentage male	24	-17.3707	0.6422	-90.6396 55.8982	0.00%	69.54%
PASI Score	16	0.2541	0.7781	-1.5133 2.0214	0.00%	67.75%
Mean age	24	-0.1233	0.8900	-1.8710 1.6243	0.00%	70.31%
Sample Size	24	0.0010	0.9177	-0.0182 0.0202	0.00%	69.23%
Number of study centres	24	All studies single centre				

Online Resource 8: Meta-regression results of MPV and psoriasis

Covariate	k	Regression coefficient – magnitude of effect size modification	p-value	95% CI (lb, ub)	Heterogeneity accounted for	Residual Heterogeneity (I^2)
Percentage male	26	5.3259	<0.0001	3.4473 7.2044	60.49%	91.24%
Duration of Psoriasis	17	-0.1430	0.0082	-0.2489 - 0.0371	29.32%	95.43%
Diabetes Prevalence	21	-16.0551	0.2558	-43.7435 11.6333	1.25%	96.67%
Psoriatic Arthritis Prevalence	10	0.6787	0.4310	-1.0106 2.3680	0.00%	88.13%
Hypertension	19	-5.5196	0.4928	-21.2918 10.2526	0.00%	96.27%
Study Design: Prospective vs retrospective (reference)	26	0.2114	0.5888	-0.5550 0.9778	0.00%	96.28%
Mean age	26	-0.0194	0.5911	-0.0901 0.0513	0.00%	96.44%
Smoking	12	0.9311	0.7022	-3.8412 5.7034	0.00%	96.34%
Cardiovascular Disease Prevalence	18	-14.9378	0.7028	-87.3219 58.8630	0.00%	97.68%
Sample Size	26	0.0001	0.9491	-0.0019 0.0020	0.00%	96.10%
PASI Score	19	-0.0005	0.9902	-0.0848 0.0838	0.00%	96.14%
Number of study centres	26	All studies single centre				
Dyslipidaemia	16	Dyslipidaemia an exclusion criteria in all studies				

Online Resource 11: Meta-regression results of MPV and PASI

Covariate	k	Regression coefficient – magnitude of effect size modification	p-value	95% CI (lb, ub)	Heterogeneity accounted for	Residual Heterogeneity (I^2)
Percentage male	19	1.1194	0.0037	0.3645 1.8742	33.45%	88.10%
Psoriatic Arthritis Prevalence	7	0.7192	0.0940	-0.1226 1.5611	33.25%	78.56%
Duration of Psoriasis	14	-0.0232	0.2223	-0.0605 0.0141	4.49%	92.41%
Smoking Prevalence	11	0.7563	0.2339	-0.4890 2.0016	5.45%	89.91%
Study Design: Prospective vs retrospective (reference)	19	-0.1596	0.2381	-0.4248 0.1056	3.63%	91.50%
PASI Score	16	0.0141	0.2876	-0.0119 0.0401	2.48%	92.70%
Diabetes Prevalence	17	-2.9177	0.5393	-12.2326 6.3973	0.00%	93.76%
Mean age	19	0.0069	0.5629	-0.0165 0.0304	0.00%	92.43%
Cardiovascular Disease Prevalence	16	-6.3091	0.5868	-29.0638 16.4456	0.00%	94.35%
Hypertension Prevalence	15	-2.2887	0.5938	-10.6992 6.1219	0.00%	92.08%
Sample Size	19	-0.0003	0.6495	-0.0014 0.0009	0.00%	92.31%
Number of study centres	18	All studies single centre				
Dyslipidaemia Prevalence	13	Dyslipidaemia an exclusion criteria in all studies				

Supplemental Figures

Online Resource 5

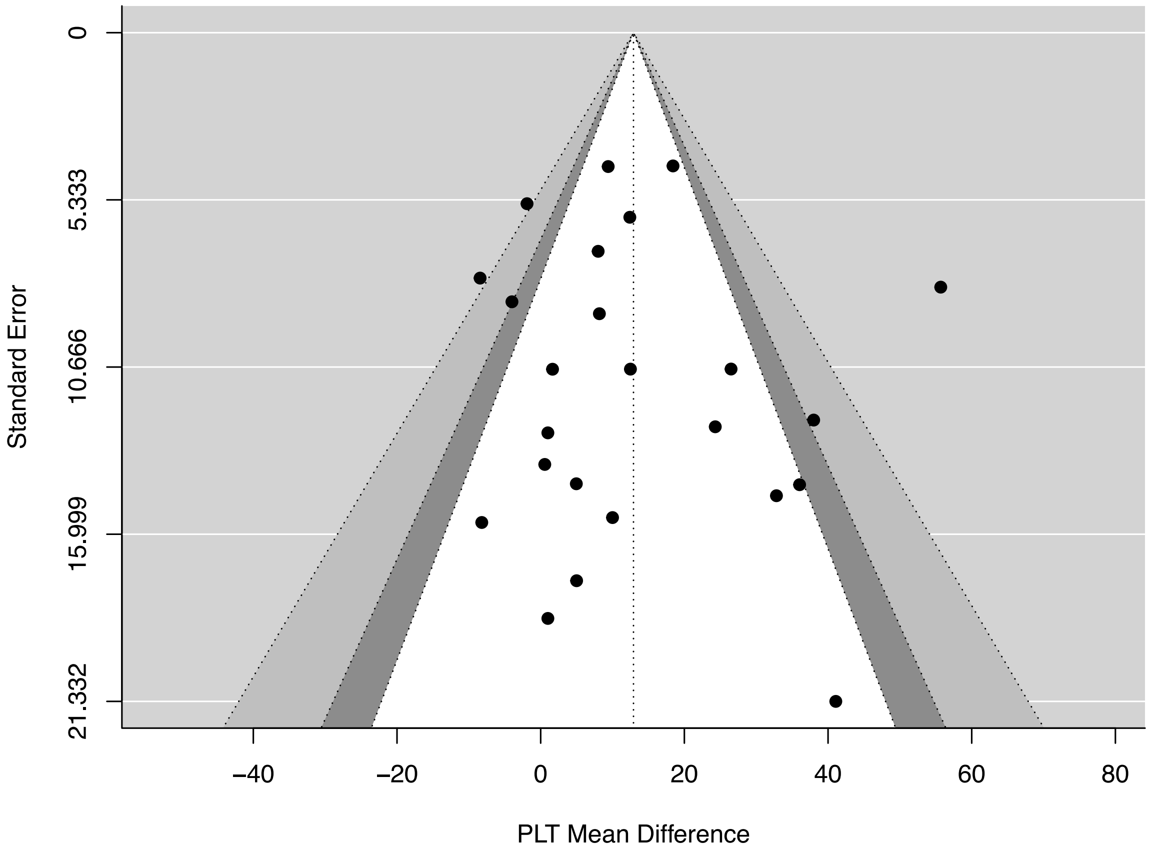

Online Resource 5: Contour enhanced funnel plot for PLT and psoriasis.

Online Resource 9

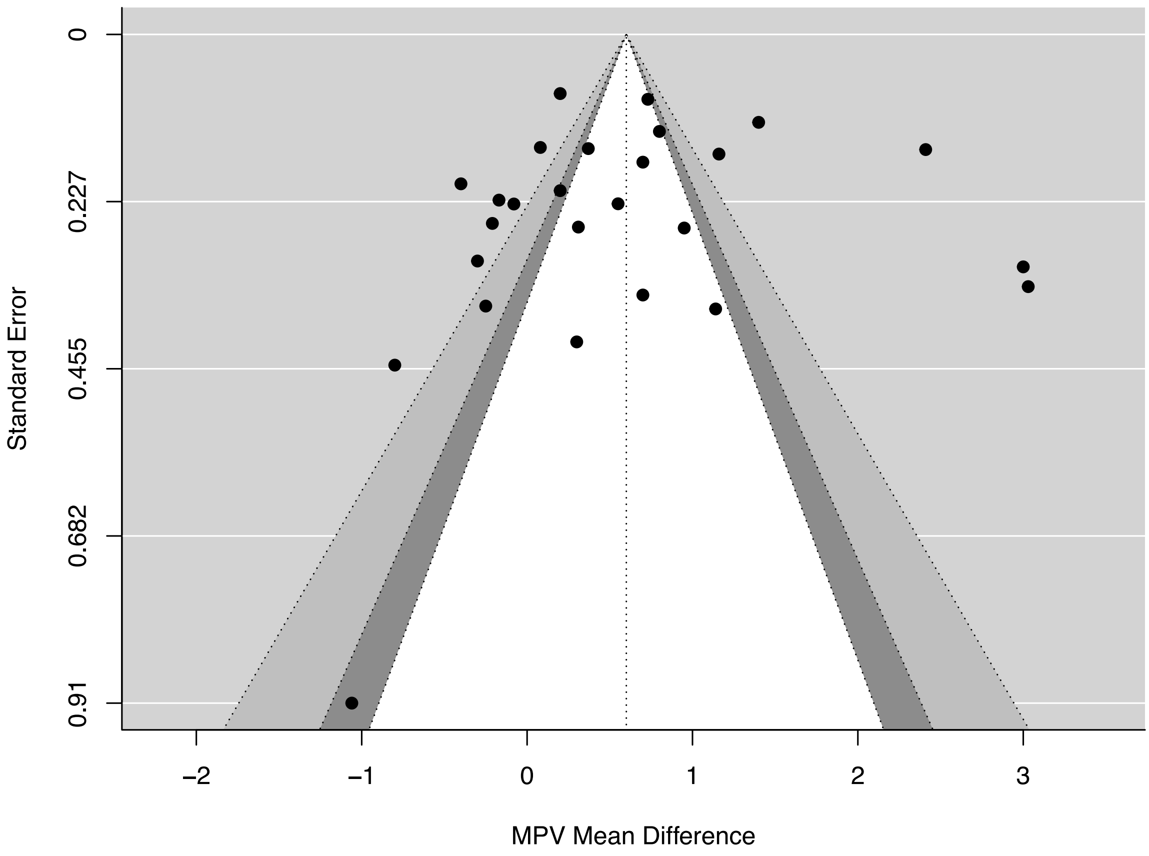

Online Resource 9: Contour enhanced funnel plot for MPV and psoriasis.

Online Resource 12

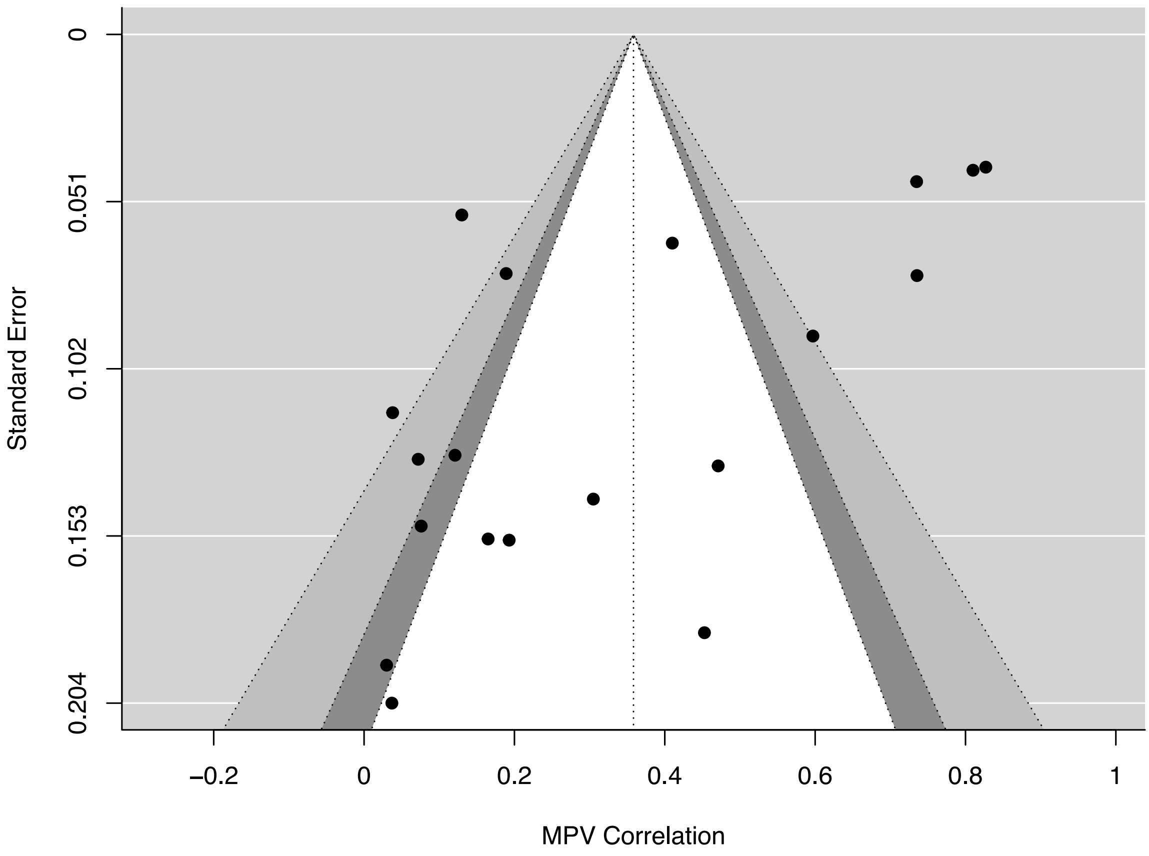

Online Resource 12: Contour enhanced funnel plot for MPV and PASI.
